# Telephone-based depression self-management in Hispanic adults with epilepsy: a pilot randomized controlled trial

**DOI:** 10.1093/tbm/ibab045

**Published:** 2021-05-08

**Authors:** Tanya M Spruill, Daniel Friedman, Laura Diaz, Mark J Butler, Keith S Goldfeld, Susanna O’Kula, Jacqueline Montesdeoca, Leydi Payano, Amanda J Shallcross, Kiranjot Kaur, Michael Tau, Blanca Vazquez, Amy Jongeling, Gbenga Ogedegbe, Orrin Devinsky

**Affiliations:** 1 Department of Population Health, NYU Grossman School of Medicine, New York, NY, USA; 2 Department of Neurology, NYU Langone Health, New York, NY, USA; 3 Center for Personalized Health, Feinstein Institutes for Medical Research, Northwell Health, Manhasset, NY, USA; 4 Institute for Excellence in Health Equity, New York University Grossman School of Medicine, New York, NY, USA

**Keywords:** Depression, Epilepsy, Disparities, Hispanic/Latinx, Mindfulness, Telehealth

## Abstract

Depression is associated with adverse outcomes in epilepsy but is undertreated in this population. Project UPLIFT, a telephone-based depression self-management program, was developed for adults with epilepsy and has been shown to reduce depressive symptoms in English-speaking patients. There remains an unmet need for accessible mental health programs for Hispanic adults with epilepsy. The purpose of this study was to evaluate the feasibility, acceptability, and effects on depressive symptoms of a culturally adapted version of UPLIFT for the Hispanic community. Hispanic patients with elevated depressive symptoms (*n* = 72) were enrolled from epilepsy clinics in New York City and randomized to UPLIFT or usual care. UPLIFT was delivered in English or Spanish to small groups in eight weekly telephone sessions. Feasibility was assessed by recruitment, retention, and adherence rates and acceptability was assessed by self-reported satisfaction with the intervention. Depressive symptoms (PHQ-9 scores) were compared between study arms over 12 months. The mean age was 43.3±11.3, 71% of participants were female and 67% were primary Spanish speakers. Recruitment (76% consent rate) and retention rates (86–93%) were high. UPLIFT participants completed a median of six out of eight sessions and satisfaction ratings were high, but rates of long-term practice were low. Rates of clinically significant depressive symptoms (PHQ-9 ≥5) were lower in UPLIFT versus usual care throughout follow-up (63% vs. 72%, 8 weeks; 40% vs. 70%, 6 months; 47% vs. 70%, 12 months). Multivariable-adjusted regressions demonstrated statistically significant differences at 6 months (OR = 0.24, 95% CI, 0.06–0.93), which were slightly reduced at 12 months (OR = 0.30, 95% CI, 0.08–1.16). Results suggest that UPLIFT is feasible and acceptable among Hispanic adults with epilepsy and demonstrate promising effects on depressive symptoms. Larger trials in geographically diverse samples are warranted.

**Implications**

**Practice:** Telephone-based depression self-management training (Project UPLIFT) is a feasible and acceptable approach to improve depressive symptoms among Hispanic adults with epilepsy.

**Policy:** Funding to support implementation of this group-based telehealth program in clinical and community settings could improve mental health equity.

**Research:** Further research is needed to test effects of UPLIFT on depressive symptoms and disease outcomes in larger, more heterogeneous samples of Hispanic adults with epilepsy.

**Lay summary** Epilepsy is a common chronic neurological disorder that involves recurrent seizures and affects people of all ages, races, and ethnicities. Many people with epilepsy experience depressive symptoms, which is related to worse health outcomes and decreased quality of life. There is an unmet need for mental health support for Hispanic people with epilepsy, who have higher rates of psychological distress than non-Hispanic whites but poor access to evidence-based, culturally sensitive interventions. Project UPLIFT is a telephone-based depression self-management program that has been shown to reduce depressive symptoms in English-speaking adults with epilepsy. The goal of this study was to test whether a culturally adapted version of UPLIFT is feasible and acceptable and reduces depressive symptoms in Hispanic men and women with epilepsy. Seventy-two participants were recruited from epilepsy clinics in New York City. The average age was 43 years, 71% of participants were female and 67% were primary Spanish speakers. A trained instructor delivered UPLIFT in English or Spanish to small groups of participants in eight weekly 1-hr telephone sessions. Feasibility was demonstrated by good recruitment, retention and session completion rates, and acceptability was demonstrated by high satisfaction ratings for UPLIFT. In addition, fewer participants assigned to UPLIFT reported high depressive symptoms at the 6-month follow-up, compared to those assigned to usual care (40% vs. 70%). If future studies confirm these findings, offering UPLIFT in clinical settings could improve the management of depression in people with epilepsy.

## INTRODUCTION

Epilepsy is a neurological disorder characterized by recurrent, unpredictable seizures and affects an estimated three million American adults of all ages, races, and ethnicities [1, 2]. Living with epilepsy involves numerous challenges that affect physical, mental, and social well-being [3]. For example, people with epilepsy (PWE) are at increased risk of physical injuries related to seizures (e.g., falls, burns) and have a higher prevalence of cognitive disorders (e.g., impaired memory and attention) and mental health disorders (e.g., depression, anxiety) than those without epilepsy [3]. These health issues, along with side effects of treatment, lifestyle impacts (e.g., limitations in driving, employment), stigma, and social isolation contribute to chronic impairments in quality of life and substantial economic burden [3, 4].

Up to 30% of PWE suffer from comorbid depression; the rate can exceed 50% in treatment-resistant epilepsy [5–7]. Depression is associated with poor seizure control, increased mortality risk and impaired quality of life [6–11]. Even mild, subclinical depressive symptoms negatively impact quality of life in PWE, independent of seizure frequency [6, 12]. Despite effective psychotherapeutic and pharmacological interventions, depression is underdiagnosed and often untreated in PWE [13, 14]. Even when provided with treatment referrals, many patients neglect to seek care due to the stigma of a mental health diagnosis, mistrust of treatments, or inadequate access (e.g., insurance, transportation) [13].

These challenges are magnified for racial and ethnic minority patients [15]. Mood disorders and serious psychological distress are more common in Hispanic PWE compared with non-Hispanic white PWE [16, 17], but treatment rates are lower [14]. Language barriers, discrimination, and disparities in healthcare access and quality contribute to depressive symptoms and pose barriers to treatment [15, 18–22]. Cultural factors play a particularly important role in the underutilization of mental health services among Hispanics. For example, Hispanics are more likely than non-Hispanic whites to prefer counseling over medication to manage depressive symptoms, but their adherence is frequently poor, due in part to low cultural competence of available counselors [23, 24]. Ethnic concordance between patients and providers and culturally sensitive depression treatments are associated with greater engagement and efficacy, particularly for Hispanic immigrants and those with limited English proficiency [25–27]. However, a 2015 survey of 4,235 practicing licensed psychologists in the USA found that only 5.5% could provide services in Spanish [28]. Increasing access to effective, culturally acceptable approaches to manage depression in Hispanic PWE represents a major opportunity to improve health outcomes and reduce disparities.

Project UPLIFT (Using Practice and Learning to Increase Favorable Thoughts) was developed to teach depression self-management skills to PWE. The program was informed by mindfulness-based cognitive therapy (MBCT), an evidence-based program for the treatment and prevention of depression [29–31], and adapted to address the needs of PWE [32, 33]. Traditional MBCT is delivered in-person in eight weekly 2 to 2.5-hr group sessions with 45 min of daily home practice. Because this intensive format poses a barrier to participation for individuals with chronic diseases, UPLIFT was designed for delivery to small groups by telephone with shorter weekly sessions (1 hr) and reduced home practice (~15 min per day). Telephone-based delivery addresses issues related to stigma and transportation, and retaining the group format builds social support, which is often limited in PWE [34, 35]. Three trials in English-speaking PWE found that UPLIFT was associated with greater reduction in depressive symptoms, lower incidence of major depressive episodes, and decreased seizure frequency and severity compared with wait-list control [33, 36–38]. However, UPLIFT has never been evaluated in Spanish-speaking PWE.

Although Hispanics are the largest U.S. minority group, there is a considerable lag in the development, evaluation, and dissemination of evidence-based, culturally tailored interventions for this population [39–41]. For example, among 69 mindfulness-based trials in the USA, only 45 reported data on non-Caucasian races and ethnicities, and of these, only 4% of participants were Hispanic/Latinx [42]. To address this unmet need, our study team conducted qualitative research from 2015 to 2016 to culturally adapt UPLIFT for Hispanic PWE [43]. Intervention materials (written and audio) were translated into Spanish and focus groups and individual interviews were conducted with Hispanic PWE to improve the materials. Adaptations included refining the Spanish translation to simplify communication of mindfulness concepts and reducing the overall literacy level of the UPLIFT workbook (both languages). Although many focus group participants expressed a preference for in-person sessions, we retained the telephone-based approach because of the enhanced access it provides for PWE and Spanish speakers. Upon finalizing the materials, we conducted a pilot randomized controlled trial of the adapted UPLIFT program. The goals of this study were to evaluate its feasibility, acceptability, and effects on depressive symptoms in Hispanic PWE over 12 months.

## METHODS

### Participants

Patients were recruited from outpatient epilepsy clinics at NYU Langone Health and Bellevue Hospital Center. Bilingual research assistants pre-screened the electronic health record (EHR) of patients with upcoming clinic appointments and obtained permission from the treating epileptologist to contact potentially eligible patients. Interested patients completed a screening interview including the Center for Epidemiological Studies Depression scale (CES-D) [44, 45]. Inclusion criteria were: ≥21 years of age; self-identified Hispanic ethnicity; fluent in English or Spanish; diagnosed with epilepsy for at least 1 year; elevated depressive symptoms (CES-D >13); willing to participate in eight weekly telephone sessions; and willing to be audiotaped. To enhance generalizability, we included participants taking antidepressant medication and adjusted for this in the analysis. Exclusion criteria were severe depressive symptoms (CES-D ≥38), active suicidal ideation, and cognitive impairment (documented on problem list in the EHR or evident during screening), as individuals with these conditions are not appropriate for this group intervention. Those excluded due to severe depression or suicidal ideation were assessed to determine safety and provided with treatment referrals. Eligible participants completed in-person baseline visits, at which time informed consent was obtained. The NYU Grossman School of Medicine and Bellevue Hospital Center Institutional Review Boards approved this study. The study was registered with clinicaltrials.gov (NCT03000725).

### Randomization and blinding

Participants were randomized to UPLIFT or usual care in cohorts of 12 at a 1:1 ratio to achieve the target intervention group size of 6. The randomization sequence was created a priori by the study statistician using a computer-generated list of random numbers. Once 12 participants with the same language preference were enrolled and completed baseline assessments, a research coordinator not involved in data collection or analysis determined group assignments for the cohort and contacted participants to inform them of assignments. The research assistants who collected follow-up data were blinded to treatment assignment. Participants could not be blinded.

### Study Arms

#### Usual care (UC)

Participants randomized to the UC arm received printed educational materials on epilepsy self-management in English or Spanish as preferred.

#### Intervention

Project UPLIFT (Using Practice and Learning to Increase Favorable Thoughts) is an 8-week, telephone-based program designed to teach skills to self-manage depressive symptoms to small groups of PWE. UPLIFT was adapted from MBCT to address barriers to in-person participation experienced by PWE [32, 33]. Participants used a free conference line to join the group sessions with their own phones (smartphones were not necessary). Sessions were live and interactive, providing education about the relationship between epilepsy and depression and time for participants to share their experiences with depression or low mood. CBT-related skills included cognitive restructuring, problem solving, behavioral activation, and relaxation training. Mindfulness activities included body scans, attention to breath, and other meditations (e.g., attention to sights, sounds). Each session was 1-hr long and was comprised of a check-in period, teaching on the week’s topic, group discussion, a skill-building exercise, and a home practice assignment (~15 min per day). Participants received workbooks and CDs with audio guides to support teaching and home practice, in English or Spanish as preferred. Sessions were co-facilitated by an MPH-level professional and a layperson with epilepsy, and supervised by a clinical psychologist.

#### Facilitator training and treatment fidelity

The lead UPLIFT facilitator was a public health professional (MPH in Community and International Health) and bilingual, native Spanish speaker who completed the 10-week UPLIFT facilitator training program. The facilitator was trained to refer participants to their physicians for any medical questions that arose during sessions. During the study, she was supervised by the PI, a licensed clinical psychologist, and participated in monthly technical assistance calls in which facilitators throughout the USA delivering UPLIFT as part of research or community-based programs discuss progress and challenges. The UPLIFT co-facilitator was a bilingual, Hispanic PWE who was trained by the study team to lead group discussions about personal experiences of epilepsy and depression during the weekly sessions. To maximize retention and compliance, research staff provided reminders to participants before each study visit and contacted those in the UPLIFT arm who missed a session. Brief individual make-up sessions (15–30 min) were provided so participants could easily re-join their group.

### Measures

#### Sociodemographic and clinical characteristics

Participants reported age, gender, race, ethnicity, education, income, marital status, and employment status. Several proxy measures of acculturation were also collected, including country of birth, years living in the USA, and primary language. Self-reported clinical factors included age of epilepsy onset, number of seizures in the past 30 days and in the past 12 months, and antidepressant medication use. Questionnaires were completed in English or Spanish as preferred. Research staff reviewed participants’ EHRs at baseline to abstract seizure types and categorized them as focal, generalized or unknown following the 2017 International League Against Epilepsy (ILAE) classification [46].

#### Feasibility

Recruitment and retention rates and UPLIFT session completion were recorded by research staff. To explore long-term adoption of intervention exercises, UPLIFT participants completed three items at the 12-month visit regarding the frequency with which they continued to engage in program practices, including: (i) formal practice (e.g., body scan, sitting meditation); (ii) informal practice (mindfulness of routine activities – walking, eating, etc.); and (iii) the 3-min breathing space. Response options were daily, frequently (≥1/week), sometimes (≥1/month), rarely (<1/month), and never.

#### Acceptability

Participants randomized to UPLIFT completed the Client Satisfaction Questionnaire (CSQ-8) [47], a widely used 8-item measure of satisfaction with health programs. Each item is rated on a 4-point scale and total scores range from 8 to 32 where higher scores indicate greater satisfaction. The CSQ-8 demonstrated good internal consistency in this study (*α* = 0.87). An open-ended question solicited feedback about participants’ experiences with UPLIFT and suggestions to improve the program to inform future revisions. The CSQ-8 was administered by a research assistant not involved in intervention delivery or other data collection to facilitate unbiased responses and maintain blinding of outcome assessments.

#### Depressive symptoms

The Patient Health Questionnaire (PHQ-9) was used to assess depressive symptoms over the past 2 weeks at each study time point. This measure is based on DSM-IV diagnostic criteria for major depression and has been validated for depression screening in PWE and in Spanish speakers [45, 48–50]. Each of the nine items is rated on a 4-point scale ranging from 0 (not at all) to 3 (nearly every day). Total scores range from 0 to 27 with recommended cutoffs for mild (5–9), moderate (10–14), moderately severe (15–19) and severe (≥20) depressive symptoms. A PHQ-9 score of 5 or more was used to define clinically significant (i.e., mild or greater) depressive symptoms, consistent with the interpretation of scores below 5 as asymptomatic [51]. Given the adverse effects of subclinical depressive symptoms in epilepsy, this cutoff reflects the importance of achieving remission [6, 12]. The research team followed a detailed safety protocol if any participant endorsed suicidal ideation on the PHQ-9, including formal assessment, consultation with the supervising psychologist and further action as needed.

### Statistical analysis

Sample characteristics were summarized overall and by study arm with means and standard deviations for continuous variables and frequencies and percentages for categorical variables. Baseline differences between the UPLIFT and UC arms were evaluated using independent samples *t*-tests and chi-squared tests for continuous and categorical variables, respectively. The feasibility of the adapted UPLIFT program was quantified as recruitment and retention rates, intervention adherence (% of sessions completed), and long-term practice rates, while acceptability was determined by intervention satisfaction (CSQ-8) scores. Research staff compiled open-ended feedback, which was reviewed by the team for selection of representative comments. Analysis of the effects of UPLIFT on depressive symptoms followed the intention-to-treat principle in which all subjects were included, regardless of intervention adherence or completion of follow-up assessments. The proportion of participants reporting elevated depressive symptoms (PHQ-9 ≥5) during follow-up was compared between arms using generalized estimating equations binomial logistic regression with an unstructured covariance matrix. We tested the effect of treatment arm (UPLIFT vs. UC), time (8 week, 6 months, and 12 months vs. baseline), and the treatment × time interaction to evaluate group differences in the proportion of participants with elevated depressive symptoms over the follow-up period. As the proportion of patients with elevated depressive symptoms did not change linearly between assessment periods, time was modeled as a categorical variable. Model 1 was adjusted for recruitment cohort and antidepressant medication use. Model 2 was further adjusted for age and education given baseline differences between the study arms.

## RESULTS

### Participant characteristics

The mean age was 42.8±11.3 years and 70.8% of the sample was female. Socioeconomic status was low, with 45.8% completing less than a high school education, 69.4% not working for pay, and 82.8% reporting <$25,000 annual household income. The mean age of epilepsy diagnosis was 17.9±14.1 years and mean duration of epilepsy was 25.5±15.5 years. Most participants (73.6%) reported having a seizure in the past 12 months (median number of seizures = 3, IQR = 1–10) and 45.8% had at least one seizure in the past 30 days. Seizure type was classified as 73.6% focal, 19.4% generalized, and 6.9% unknown. The mean PHQ-9 score at baseline was 8.4±4.7 and 80.6% of participants had scores ≥5. Almost one-third of participants (31.9%) were taking antidepressant medication.

Most participants (72.1%) were born outside the USA. The most common countries of origin were Mexico (28.6%), Ecuador (22.4%), Dominican Republic (20.4%), and Puerto Rico (18.4%); the remaining 10.1% were from Colombia, El Salvador, and Peru. The foreign-born participants had lived in the USA for a mean of 25.7±12.6 years. Two-thirds of participants chose to complete UPLIFT in Spanish and one-third in English. There were no significant differences in baseline depressive symptoms or clinical factors by language, but Spanish-speakers had lower income (*p* = .046) and education levels (*p* = .010) than English-speakers.

Table 1 presents sample characteristics by study arm. Participants randomized to UPLIFT were older and less likely to be married than those randomized to UC. They also had somewhat higher education, were less likely to be employed, and were more likely to be taking antidepressant medication. Mean baseline PHQ-9 scores were somewhat higher in the UC versus UPLIFT arm, but the proportion of participants in each arm with scores ≥5 was identical.

**Table 1:** Demographic and baseline characteristics

	Total (*n* = 72)	UPLIFT (*n* 36)	Usual care (*n* 36)	*p*-value
Age	43.3 (11.3)	47.0 (11.4)	39.6 (10.0)	.005
Male	21 (29.2%)	10 (27.8%)	11 (30.6%)	.795
Education				.100
Less than high school	33 (45.8%)	13 (36.1%)	20 (55.6%)	
High school graduate	33 (45.8%)	21 (58.3%)	12 (33.3%)	
College graduate	6 (8.3%)	2 (5.6%)	4 (11.1%)	
Not working for pay	50 (69.4%)	28 (77.8%)	22 (61.1%)	.125
Household income*				.865
<$25,000	53 (82.8%)	27 (81.8%)	26 (83.9%)	
$25,000-$49,999	8 (12.5%)	4 (12.1%)	4 (12.9%)	
≥$50,000	3 (4.7%)	2 (6.1%)	1 (3.2%)	
Married/coupled	23 (31.9%)	7 (19.4%)	16 (44.4%)	.023
Primary language Spanish	48 (66.7%)	24 (66.7%)	24 (66.7%)	1.00
Antidepressant medication use	23 (31.9%)	14 (38.9%)	9 (25.0%)	.206
Baseline PHQ-9 score	8.4 (4.7)	8.1 (4.7)	8.7 (4.7)	.618
Baseline PHQ-9 ≥5	58 (80.6%)	29 (80.6%)	29 (80.6%)	1.00
≥1 seizure in past 12 months	53 (73.6%)	26 (72.2%)	27 (75.0%)	.789

Continuous data represented as mean (*SD*) and categorical data presented as *n* (%).

*Excludes 8 participants who declined to report income.

### Feasibility

#### Recruitment and retention

**Fig 1:**
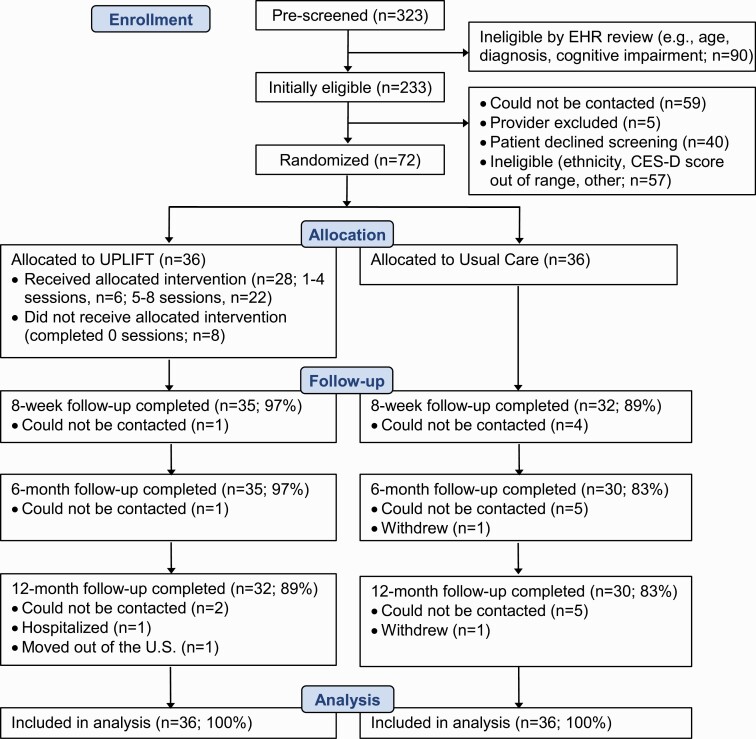
Consolidated Standards of Reporting Trials (CONSORT) diagram.

Figure 1 shows the Consolidated Standards of Reporting Trials (CONSORT) diagram. From October 2016 to April 2018, 323 patients were pre-screened using medical records and 233 were initially eligible based on age and diagnosis. Of 169 patients whose physicians approved contact and who were successfully reached, 129 (76.3%) wished to participate and were screened for the study, of whom 57 (44.2%) were ineligible, and 72 (55.8%) were eligible and enrolled. Retention rates were 93% at 8 weeks, 90% at 6 months and 86% at 12 months, and were similar in English- and Spanish-speaking participants. As shown in Fig. 1, retention was somewhat higher in the UPLIFT versus UC arm.

#### Intervention adherence

The median number of UPLIFT sessions completed was 6 of 8 (IQR, 1–8) and 61% of participants (75% English-speaking vs. 54% Spanish-speaking, *p* = .23) completed four or more sessions, considered the minimum effective dose for in-person MBCT [52]. Eight participants (22%) did not complete any sessions. At the 12-month follow-up, 35% of UPLIFT participants reported continued engagement in informal mindfulness practice (e.g., mindfulness of routine activities – walking, eating) at least once per week. Fewer reported engaging in the 3-min breathing space (23%) or formal mindfulness practices (e.g., body scan, sitting meditation; 15%) at least once per week.

### Acceptability

Of the 28 participants who completed at least one UPLIFT session, 23 (82%) completed the CSQ-8. The vast majority (96%) reported being mostly or very satisfied with UPLIFT overall (vs. indifferent or dissatisfied) and would recommend the program to a friend (definitely, 74%; think so, 26%). Most participants (83%) indicated that UPLIFT helped them deal more effectively with their problems, while 65% indicated that UPLIFT met most or all of their needs. The mean CSQ-8 score was 27.0±3.7 and 83% of participants reported scores ≥24, indicating high satisfaction. CSQ-8 scores were similar in English- and Spanish-speaking participants (27.2 vs. 26.8, *p* = .78).

High satisfaction with UPLIFT was also reflected in open-ended participant feedback, with representative quotes in Table 2. The most common themes were the value of connecting with other PWE and improved ability to manage negative thoughts and emotions. The two participants who stopped attending after two sessions were positive about the program content but found the group format challenging. Several participants commented on the advantages of the phone-based approach, but two indicated they would have preferred to have at least one in-person session. Table 2 includes other suggestions to improve the program, including longer sessions, more sessions and assistance with home practice. The UPLIFT facilitator also provided feedback after completing intervention delivery for all study cohorts. Overall, Spanish-speaking participants had more difficulty understanding mindfulness concepts than English-speakers and would benefit from further simplifying terminology in the workbook. The facilitator also suggested that future UPLIFT groups in Spanish would benefit from additional culturally relevant examples and metaphors (e.g., using perceived stigma in thought monitoring exercises instead of more general prompts).

**Table 2:** Feedback from UPLIFT participants

Positive experiences	“What I liked the most was to know that I am not the only one going through this problem, to know that there are other people that have depression, and to listen to others’ stories.”
	“I enjoyed the program because even though we were not able to see each other we were able to talk to one another about different topics, about different aspects of epilepsy.”
	“I felt that by participating I could get help to control my mood and my thoughts about myself. I have learned to value myself better.”
	“With the program it helped me to open up, and speak freely with my problem. It has made me a different person.”
	“I enjoyed the program because it helped me to remember the negative events in my life and provided me a way to manage my negative thoughts.”
Challenges	“The way [facilitator] communicates makes me feel like I can talk. She inspires trust and made me feel safe. … On the other hand, it is difficult to speak personal matters on the phone. I feel better when I am in groups in person.”
	“Some things were a little difficult to understand. My mother had to help me with some words from the book.”
	“I think some people were very depressive. It was very strong, that was one of the reasons why I stopped calling.”
Suggestions for improvement	“I think it was well structured but I would prefer to have an in-person session, at least once.”
	“Try to find a way for participants to do their homework easier. … Maybe sending a reminder will help. Maybe using an app.”
	“Something that will be helpful is to add more time to sessions since one hour is not enough. Also, I think it will be helpful if you guys add more sessions into the program.”

### Changes in depressive symptoms

**Fig 2:**
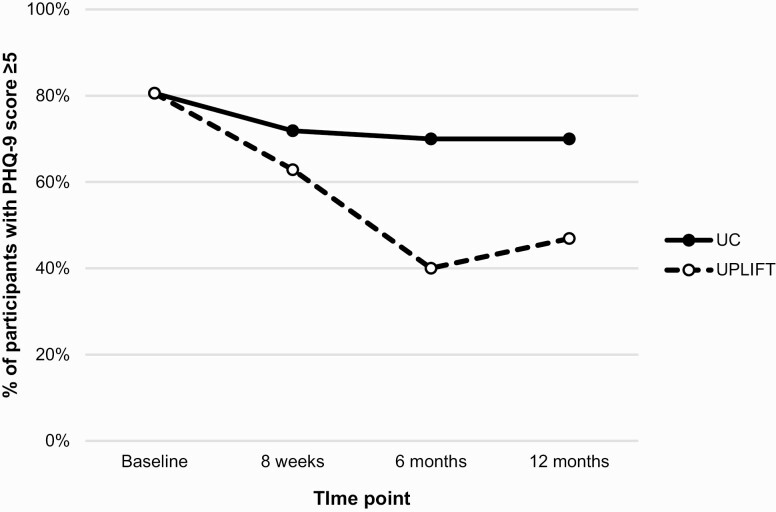
Proportion of participants with mild or greater depressive symptoms (PHQ-9 ≥5) over 12 months by study arm.

As shown in Table 3, small reductions in PHQ-9 scores were observed in both arms over the follow-up period, with the greatest between-group difference occurring at 6 months (5.8±4.6 [UPLIFT] vs. 7.1±4.7 [UC], *p* = .288). The proportion of participants reporting clinically significant depressive symptoms (PHQ-9 ≥5) was lower in the UPLIFT versus UC arm throughout follow-up (63% vs. 72% at 8 weeks [*p* = 0.432]; 40% vs. 70% at 6 months [*p* = 0.016]; 47% vs. 70% at 12 months [*p* = 0.065]; Fig. 2). Table 4 shows results of generalized estimating equations adjusted for cohort, age, education, and antidepressant medication use. The results indicate that the beneficial effect of UPLIFT was greatest at 6 months (OR = 0.24, 95% CI, 0.06–0.93) and somewhat reduced at 12 months (OR = 0.30, 95% CI, 0.08–1.16).

**Table 3:** Mean (*SD*) PHQ-9 scores over time by study arm

Time point	Total	UPLIFT	Usual care	SMD	*p*-value
Baseline (*n* = 72)	8.4 (4.7)	8.1 (4.8)	8.7 (4.7)	.123	.607
8 weeks (*n* = 67)	7.9 (5.0)	7.6 (4.8)	8.2 (5.1)	.112	.648
6 months (*n* = 65)	6.4 (4.7)	5.8 (4.6)	7.1 (4.7)	.266	.288
12 months (*n* = 62)	7.1 (5.5)	6.9 (6.3)	7.2 (4.5)	.054	.833

*SMD* standardized mean difference.

**Table 4:** Intervention effects (UPLIFT vs. Usual care) on the rate of mild depressive symptoms (PHQ-9 ≥5) over time

	Model 1*		Model 2**	
Predictor	OR (95% CI)	*p*-value	*OR* (95% CI)	*p*-value
Intervention	0.89 (0.27–2.89)	.840	0.80 (0.24–2.70)	.720
8 weeks	0.59 (0.21–1.69)	.327	0.58 (0.19–1.71)	.320
6 months	0.52 (0.23–1.18)	.119	0.51 (0.22–1.18)	.115
12 months	0.53 (0.24–1.19)	.126	0.52 (0.22–1.20)	.123
Intervention * 8 weeks	0.64 (0.14–2.85)	.557	0.64 (0.13–3.08)	.579
Intervention * 6 months	0.26 (0.07–0.95)	.042	0.24 (0.06–0.93)	.038
Intervention * 12 months	0.32 (0.09–1.16)	.083	0.30 (0.08–1.16)	.080

*Model 1 – Adjusted for recruitment cohort and antidepressant medication use.

^**^Model 2 – Adjusted for covariates in model 1 plus age and education status.

## DISCUSSION

Results of this randomized controlled trial demonstrate that telephone-based depression self-management training is feasible and acceptable among Hispanic adults with epilepsy and is associated with greater improvement in depressive symptoms compared to usual care. This benefit was observed for participants who completed the UPLIFT program in Spanish or English and was independent of age, education and antidepressant medication use. Although changes in continuous PHQ-9 scores were relatively small, the difference in the proportion of UPLIFT and usual care participants with mild or greater depressive symptoms at 6 months (40% vs. 70%, respectively) was clinically meaningful. Subclinical depressive symptoms are common in epilepsy and affect quality of life to a similar degree as major depressive episodes, but are less likely to be treated [12, 13]. Therefore, effective self-management programs address a gap in care and have the potential to significantly improve mental health and epilepsy outcomes [53].

The rate of elevated depressive symptoms remained lower in the UPLIFT versus the usual care arm at 12 months (47% vs. 70%), but the difference was no longer statistically significant (*p* = .08). This may reflect decay of the intervention effect or lack of statistical power. Still, this finding is promising given the brief intervention period and relatively low rates of continued practice of program exercises. The only other trial of UPLIFT that followed participants for 12 months similarly found that effects were largely maintained [37]. Meta-analyses have also demonstrated sustained effects of mindfulness-based interventions on depressive symptoms and depression relapse [29–31], suggesting that such programs may promote lasting changes in emotion regulation skills. The importance of the frequency and type of home practice in achieving such effects remains unclear and should be investigated in future studies [54, 55]. Whether booster sessions or other forms of support beyond the intervention period (e.g., reminders, mindfulness apps) improve long-term adherence to program exercises, enhance reductions in depressive symptoms and prevent rebound should also be explored.

Our sample was comprised primarily of young and middle-aged Spanish-speaking immigrants from low socioeconomic backgrounds. Results demonstrate that the UPLIFT program is feasible and acceptable in this population. More than three-quarters of patients approached were interested in participating and consented to screening, and more than half of those screened were eligible and enrolled. Post-intervention retention (93%) was comparable or higher than in prior UPLIFT trials [33, 36], and the 86% retention at 12 months is noteworthy given the residential mobility of the Hispanic population in NYC. Session completion was comparable or higher than in trials of in-person, online, and telephone-based mindfulness programs, including trials of UPLIFT in non-Hispanic PWE [33, 36, 56, 57]. This is an important finding given that many focus group participants expressed a preference for in-person sessions in our prior qualitative research [43]. During recruitment for the trial, research staff emphasized the advantages offered by UPLIFT to prospective participants, particularly the unique opportunity to interact with other Hispanic PWE. Post-intervention feedback indicated that many participants did benefit from the remote group-based format, and overall satisfaction ratings were high. Evidence of the feasibility and acceptability of UPLIFT among Hispanic PWE are key findings given that transportation and limited availability of culturally competent providers are significant barriers to high quality care [58]. The need for telehealth approaches has become even more urgent given the COVID-19 pandemic, which has exacerbated access barriers while contributing to psychological distress and social isolation. Because minority communities have been disproportionately affected, programs like UPLIFT could improve mental health equity [59].

Despite a rigorous cultural adaptation process, our UPLIFT facilitator reported that Spanish-speaking participants had more difficulty understanding mindfulness concepts than English speakers. This could reflect lower education, health literacy, and acculturation levels, which are more common among immigrants versus U.S.-born Hispanics [22, 60]. It is also possible that the Spanish translation was not optimally suited to Hispanic participants from different geographical regions. Continued efforts to improve the linguistic and cultural sensitivity of UPLIFT for Spanish speakers are needed. We also found that session completion rates were somewhat lower in the Spanish vs. English groups, though satisfaction ratings were similar. Given that socioeconomic status was lower in Spanish- versus English-speaking participants, logistical barriers (e.g., related to work, caregiving) may have interfered with their ability to attend sessions despite valuing the program. Future studies should explore the usefulness of strategies to enhance engagement and adherence, such as individual orientation sessions to allow the facilitator to establish a connection with each participant and address questions, concerns, and potential barriers prior to the start of the program.

Strengths of this study include recruitment of a socioeconomically disadvantaged and culturally diverse group of Hispanic PWE and high retention rates through 12 months of follow-up. Our target population is a vulnerable, hard-to-reach group that is often underrepresented in research, particularly studies of mental health interventions. Thus, these findings make an important contribution to the literature. However, our results must be interpreted in the context of several limitations. The sample size was relatively small and recruitment was limited to New York City, where the Hispanic population differs from other parts of the USA. Future trials should include culturally and geographically diverse samples of Hispanic PWE. We did not assess social desirability, which could have influenced satisfaction ratings. In addition, because we compared UPLIFT to usual care, we cannot exclude that nonspecific treatment effects (e.g., attention, expectation of benefit) explained the findings. We also cannot differentiate the relative importance of UPLIFT components (i.e., mindfulness and cognitive skills, social support). A recent 3-arm trial in which UPLIFT was compared to wait-list control and an epilepsy education program with a matched format (eight weekly group phone sessions) found that the two interventions achieved similar improvements in depressive symptoms [37]. Comparing UPLIFT to carefully designed active control conditions that offer education and/or support in future trials will help to identify its active ingredients and ensure that any investment in implementing the program is justified.

## CONCLUSIONS

In this study, Project UPLIFT, a telephone-based depression self-management intervention developed for people with epilepsy, was evaluated in the Hispanic population for the first time. The culturally adapted UPLIFT program was found to be feasible and acceptable, and was associated with lower rates of clinically significant depressive symptoms over 12 months compared with usual care. These findings indicate that UPLIFT is a promising approach to address the urgent need for mental health support in this underserved population. Future studies should continue to refine the program to maximize its cultural sensitivity and evaluate its efficacy in larger trials with more diverse samples.
